# Acetylcholinesterase immobilization on a natural thin membrane for stable biocatalyst design

**DOI:** 10.55730/1300-0527.3904

**Published:** 2026-06-09

**Authors:** Sinem ÖZTÜRK, Ceyhun IŞIK, Mustafa TEKE

**Affiliations:** Department of Chemistry, Faculty of Science, Muğla Sıtkı Koçman University, Muğla, Turkiye

**Keywords:** AChE, immobilization, natural membrane support, biocatalysis

## Abstract

This study involved the immobilization of acetylcholinesterase (AChE) onto a natural thin membrane extracted from the inner epidermal layer of *Allium cepa*, with the aim of enhancing the enzyme’s stability, activity, and reusability for potential biotechnological applications. The membrane, composed of biocompatible polymers and naturally occurring functional groups, was used as a sustainable support in a combined strategy involving adsorption followed by glutaraldehyde-mediated cross-linking. Immobilization conditions were optimized by evaluating key factors such as enzyme concentration, membrane quantity, cross-linker dosage, and contact time to achieve maximal catalytic efficiency. Structural and morphological changes on the membrane surface were assessed via scanning electron microscopy and infrared spectroscopy, confirming successful enzyme binding and alterations indicative of interaction. The immobilized enzyme exhibited enhanced tolerance to pH and temperature variations when compared to its free form. While both free and immobilized enzymes showed peak activity at 30 °C, the immobilized enzyme retained approximately 80% of its activity at 40 °C, whereas the free enzyme retained only around 50%. A reusability assessment demonstrated that the immobilized system preserved over 60% of its initial activity after 10 consecutive cycles. Moreover, storage stability tests revealed that the immobilized enzyme maintained nearly 40% of its catalytic function after 30 days at room temperature. These results highlight the effectiveness of the onion membrane as a green and low-cost platform for enzyme immobilization, offering considerable promise for applications in industrial biocatalysis, biosensing, and diagnostic technologies.

## 1. Introduction

Enzymes are highly specific and efficient biological catalysts widely used in various fields, including biochemistry, food processing, textiles, and pharmaceuticals [1–3]. However, their practical applications are often limited by sensitivity to environmental conditions, difficulties in recovery from reaction media, and lack of reusability [4–6]. To address these challenges, enzyme immobilization has been extensively explored as an effective strategy [7]. Enzyme immobilization is a technique in which enzyme molecules are attached to or entrapped within a water-insoluble solid support using physical or chemical methods [8]. Immobilization involves attaching or entrapping enzymes onto solid supports using physical or chemical methods, thereby improving their stability, reusability, and operational performance [9,10]. The properties of the support material play a critical role in determining the overall efficiency of immobilization systems, and both synthetic and naturally derived polymers have been widely investigated for this purpose [11–13].

Several biologically derived membranes, including those obtained from bamboo, eggshells, and various plant tissues, have attracted increasing interest as enzyme immobilization platforms due to their natural composition of proteins, lipids, and carbohydrates [14–18]. These materials provide a biocompatible environment that facilitates efficient enzyme immobilization. The inner epidermis of the onion is a complex extracellular matrix rich in polymers such as polygalacturonic acid, proteins, lignin, hemicelluloses, and phenolic compounds [19]. Its cellulose-based microfibrillar structure makes the onion membrane particularly suitable for enzyme immobilization. This organized architecture provides a biocompatible microenvironment that supports enzyme attachment and stability [20,21]. In addition, the inherent robustness of the membrane contributes to its mechanical strength, offering a stable surface for immobilization and enhancing catalytic performance [15,22].

Acetylcholinesterase (AChE, EC 3.1.1.7) is a key enzyme responsible for the hydrolysis of acetylcholine (ACh) into choline and acetate, thereby regulating neurotransmission in both central and peripheral nervous systems [23]. This function is essential for maintaining proper neuronal signaling and preventing overstimulation of cholinergic receptors. Dysregulation of AChE activity has been associated with neurodegenerative disorders, particularly Alzheimer’s disease, which is characterized by impaired cholinergic function and cognitive decline [24,25]. Due to its critical biological role, AChE is widely used not only as a therapeutic target but also in applications such as pesticide detection and biosensor development [26,27]. In recent years, immobilization of AChE onto various support materials, including nanoparticles, polymeric membranes, and sol–gel matrices, has attracted significant attention for enhancing enzyme stability and reusability in industrial and analytical applications [28–31].

In the present study, AChE was immobilized onto a natural thin membrane obtained from the inner epidermal layer of *Allium cepa*, a readily available and low-cost plant source. Although onion membranes have previously been explored as enzyme immobilization supports, their application in AChE immobilization remains limited, and the combined effects of adsorption and cross-linking strategies on such natural matrices have not been sufficiently investigated. The present work addresses this gap by employing a dual immobilization approach that integrates adsorption with glutaraldehyde-mediated cross-linking, aiming to enhance both enzyme loading and structural stability. Unlike conventional immobilization platforms such as nanoparticles, nanofibers, and aerogels, which often require complex synthesis procedures and higher production costs, the onion membrane offers a sustainable, biodegradable, and easily accessible alternative with inherent functional groups that facilitate enzyme attachment. Furthermore, the present study provides a comprehensive evaluation of the immobilized system by systematically investigating key immobilization parameters and assessing catalytic performance, kinetic behavior, thermal and pH stability, storage stability, and reusability. The results demonstrate that, despite a moderate reduction in substrate affinity, the immobilized AChE exhibits enhanced stability and operational durability. By combining a natural support material with a dual immobilization strategy and presenting a detailed comparative analysis of enzyme performance, our study contributes to the development of cost-effective and environmentally friendly immobilization platforms for biotechnological and diagnostic applications.

## 2. Experimental

### 2.1. Materials

AChE (EC 3.1.1.7) from *Electrophorus electricus*, along with 5,5′-dithiobis(2-nitrobenzoic acid) (DTNB), acetylcholine chloride, acetylthiocholine iodide (ATChI), glutaraldehyde (GA), Coomassie Brilliant Blue G-250, and bovine serum albumin (BSA) were obtained from Sigma-Aldrich (USA). Fresh *Allium cepa* bulbs used for membrane preparation were sourced from local vendors in Muğla, Türkiye.

### 2.2. AChE activity assay

The catalytic activity of both free and immobilized AChE was determined spectrophotometrically based on a modified Ellman assay. The reaction system included 150 μL of 2 mM DTNB, 50 μL of 0.1 mM ATChI, and 540 μL of 50 mM sodium phosphate buffer (pH 8.0). AChE stock solution was prepared by dissolving 125 μg of enzyme in 10 mL of the same buffer, and 10 μL of this solution was introduced into the reaction to initiate the hydrolysis. The total reaction volume was adjusted to 750 μL and, after 1 min of incubation, absorbance was recorded at 412 nm.

For immobilized AChE activity measurements, 7.5 mg of onion membrane was used, which had been pretreated with 1 mL of the AChE solution for 10 min and subsequently crosslinked with 1% GA. One unit of AChE activity was considered the enzyme quantity necessary to catalyze the hydrolysis of 1.0 μmol of acetylcholine per minute under standard assay conditions (37 °C, pH 8.0), resulting in the formation of acetate and choline.

### 2.3. Protein determination

The concentration of protein was quantified using Bradford’s method, with measurements taken spectrophotometrically at 595 nm [32].

### 2.4. Preparation of the onion membrane for immobilization

Fresh bulbs of *Allium cepa* L. were sourced from a local vendor and stored at room temperature prior to their use in immobilization studies. The inner epidermis was prepared by cutting the onions in half and carefully separating the bulb scales. The membrane from the inner epidermis was gently peeled off the outer fleshy scales and thoroughly rinsed several times with distilled water to eliminate surface contaminants. The cleaned membranes were then cut into small pieces and stored at 4 °C in a refrigerator for subsequent use.

### 2.5. Immobilization of AChE onto onion membrane

The immobilization of AChE onto onion membrane was achieved through adsorption and cross-linking techniques. Key parameters optimized during the immobilization process included AChE concentration (0.25–1.5 μg/mL), onion membrane amount (5–15 mg), GA concentration (1–5% v/v), and adsorption time (10–30 min).

To immobilize AChE via adsorption, 1 mL of AChE solution was applied to 7.5 mg of onion membrane. The mixture was agitated on a shaker at 25 °C for 15 min to facilitate enzyme attachment. In the subsequent cross-linking step, 40 μL (1%) of GA was added to the mixture, followed by another 15 min of agitation under the same conditions. Afterward, the onion membrane was separated from the solution using a micropipette, and the immobilized onion membrane was thoroughly rinsed with distilled water multiple times to remove any unreacted GA.

### 2.6. Structural and morphological characterization of onion membrane and AChE immobilized onion membrane

Structural and chemical alterations on the onion membrane surface resulting from the immobilization process were examined using Fourier transform infrared spectroscopy (FTIR). Spectral data were obtained with a Nicolet iS-5 instrument (Thermo Scientific) to identify variations in functional groups. To analyze the morphology of onion membrane and AChE-immobilized onion membrane, prior to scanning electron microscopy (SEM) analysis a thin gold layer was deposited onto the sample surfaces using a sputter coater operated at 15 mA for 1 min to enhance conductivity. SEM images were captured at varying magnifications using a JEOL JSM 7600F.

### 2.7. Determination of enzymatic properties of free AChE and AChE immobilized on onion membrane

The catalytic behavior of both free AChE and the immobilized form on onion membrane was evaluated under different experimental conditions. The temperature-dependent activity of free and immobilized AChE was assessed using a colorimetric assay, with measurements performed from 20 °C to 65 °C to determine the optimal operating temperature. Thermal stability was evaluated by incubating the enzymes at 30 °C to 60 °C for 60 min, followed by activity assessments.

The optimal pH for enzymatic activity was determined by conducting measurements in 50 mM buffer systems, including sodium phosphate, citrate, Tris-HCl, and sodium acetate, adjusted to pH values from 3.0 to 10.0. To evaluate pH stability, both free and immobilized AChE were preincubated in buffers ranging from pH 4.0 to 9.0 for 1 h, followed by activity assays.

The operational reusability of the immobilized enzyme system was assessed through successive activity measurements, continuing until the residual activity dropped below 50% of the initial level. After each cycle, the membrane support was carefully retrieved with forceps, rinsed thoroughly three times using 50 mM sodium phosphate buffer (pH 8.0), and transferred into a fresh reaction mixture containing the ATChI substrate.

Long-term stability was evaluated by incubating both free and immobilized AChE in distilled water at 25 °C for 30 days, after which residual activity was measured. Enzymatic activity was measured at predefined intervals to monitor stability and retention of activity over the storage period.

Kinetic parameters, including the Michaelis–Menten constant (*K*_m_) and the maximum velocity (*V*_max_), were calculated for both free and immobilized AChE by measuring enzyme activity at different substrate concentrations (0.2–2.0 mM ATChI).

## 3. Results and discussion

### 3.1. Optimization of AChE immobilization on onion membrane

The immobilization strategy employed in the present study involves a combination of adsorption and glutaraldehyde-mediated cross-linking, each contributing distinct roles to the overall performance of the system. The onion membrane serves as a biocompatible support matrix, providing a porous and cellulose-rich structure with functional groups such as hydroxyl, carboxyl, and phenolic moieties that facilitate enzyme attachment. In the initial stage, adsorption enables rapid and reversible binding of AChE onto the membrane surface through noncovalent interactions, including hydrogen bonding and electrostatic forces. This step ensures effective distribution of the enzyme across the support material. Subsequently, glutaraldehyde-mediated cross-linking enhances the stability of the immobilized enzyme by forming covalent bonds between enzyme molecules and/or between the enzyme and the support surface. This process increases structural rigidity and reduces enzyme leaching during repeated use. The combined use of adsorption and cross-linking provides a synergistic effect, where adsorption ensures efficient enzyme loading while cross-linking stabilizes the enzyme structure and improves operational durability. This dual approach contributes to the improved stability, reusability, and overall performance of the immobilized AChE system observed in the present study.

The optimization parameters for AChE immobilization on onion membrane were assessed by measuring the specific activity of the immobilized enzyme, and the results are summarized in Table 1.

The specific activity of AChE immobilized on the onion membrane exhibited a rising trend with increasing enzyme concentrations, reaching its peak value of 1.70 × 10^−3^ U/mg protein at 12.5 × 10^−4^ mg/mL. However, a marginal decline in activity was detected at higher concentrations, such as 1.52 × 10^−3^ U/mg protein at 15.0 × 10^−4^ mg/mL. These results indicate that enhanced enzyme loading initially promotes better surface interaction and immobilization efficiency. Yet, beyond a critical concentration, the available binding sites on the membrane may become saturated, possibly causing steric hindrance or aggregation effects that reduce effective enzyme activity.

Increasing the amount of onion membrane led to a gradual enhancement in the specific activity of the immobilized enzyme, with the highest activity observed at 12.5 mg of support, reaching 2.88 × 10^−3^ U/mg protein. Increasing the onion membrane quantity to 15 mg led to a slight decrease in activity, reducing it to 1.51 × 10^−3^ U/mg protein. The activity enhancement up to 12.5 mg is likely due to the increased surface area, which provides more binding sites for effective enzyme immobilization. The observed decline in activity at higher support quantities could be attributed to a reduced enzyme density on the membrane surface, which may limit enzyme–substrate interactions and lower overall catalytic performance.

A progressive increase in the specific activity of AChE immobilized on onion membrane was observed with increasing adsorption time, reaching a peak value of 1.27 × 10^−3^ U/mg protein at 15 min. However, extending the contact duration beyond this point led to a marked reduction in activity, declining to 0.34 × 10^−3^ U/mg protein at 30 min. This reduction is likely due to partial desorption of the enzyme or conformational instability induced by prolonged exposure to the immobilization environment. Therefore, an adsorption period of 15 min was determined to be optimal for achieving effective enzyme immobilization.

Increasing GA concentration enhanced the specific activity of AChE immobilized on the onion membrane, with the highest activity (1.10 × 10^−3^ U/mg protein) observed at 3% GA. Beyond this concentration, the activity sharply declined, dropping to 0.20 × 10^−3^ U/mg protein at 5% GA. The initial rise in activity is likely due to efficient cross-linking, which strengthens enzyme stability and promotes firm attachment to the onion membrane surface. However, excessive cross-linking at higher GA concentrations likely caused conformational changes or structural rigidity in the enzyme, thereby hindering its catalytic activity.

Similar studies on the determination of optimal immobilization conditions have been previously reported in the literature. In one of our earlier studies, in which AChE was immobilized on PVA/Zn nanofiber membranes, the optimal immobilization parameters were identified as 1.25 mg/mL AChE concentration, 2% GA concentration, 7.5 mg carrier amount, and 10 min of adsorption time [30]. Additionally, in a study in which AChE was immobilized on Fe_3_O_4_@MXene@PEI aerogel, the optimal conditions were determined to include 20% GA concentration, a cross-linking time of 2 h, and an immobilization time of 2.5 h [33]. Furthermore, in a study in which AChE was immobilized on chitosan/PVA nanofibers, the optimal GA concentration was reported to be 1% [34]. Likewise, a study on AChE immobilization using polyvinyl alcohol/bovine serum albumin nanofibers identified the optimal enzyme concentration as 1 μg of AChE per 1 mg of carrier [35].

Table 2 presents the activity characteristics of free AChE and AChE immobilized on onion membrane, emphasizing the variations in activity, protein amount, specific activity, bound protein, and specific activity yield between the two enzyme forms.

The total activity of free AChE was 3.50 × 10^−6^ U, while the immobilized AChE exhibited reduced activity of 1.26 × 10^−6^ U. Such a decrease in enzymatic activity after immobilization is commonly reported in the literature and is generally attributed to factors such as partial enzyme inactivation, steric hindrance, and diffusion limitations that restrict substrate accessibility to the active site. Similar reductions in activity have been observed in AChE immobilization studies using various support materials, including nanoparticles, nanofiber matrices, and hybrid systems [30,36,37]. The protein content for free AChE was 10.50 × 10^−4^ mg, whereas it decreased to 4.42 × 10^−4^ mg after immobilization, indicating that only a fraction of the enzyme was successfully bound to the support. The specific activity of free AChE was 3.33 × 10^−3^ U/mg protein, while the immobilized enzyme showed a slightly lower value of 2.85 × 10^−3^ U/mg protein. This reduction suggests that immobilization may impose structural constraints on the enzyme, potentially affecting substrate enzyme interactions or altering the conformation of the active site. Despite this decrease, the percentage of bound protein (42.09%) and the specific activity yield (85.59%) indicate that a substantial portion of the immobilized enzyme retained its catalytic functionality. This suggests that onion membrane provides a supportive microenvironment that preserves enzyme activity while enabling effective immobilization. The presence of functional groups such as carboxyl, hydroxyl, and phenolic moieties likely facilitates enzyme attachment through electrostatic interactions and hydrogen bonding, contributing to the stabilization of the enzyme structure.

Overall, although a moderate decrease in activity was observed, this trade-off is typical in immobilization systems and is compensated for by the enhanced stability, reusability, and operational performance of the immobilized enzyme. These findings demonstrate that onion membrane is an effective support material that balances catalytic efficiency with improved functional stability.

### 3.2. FTIR analysis

**Figure 1:**
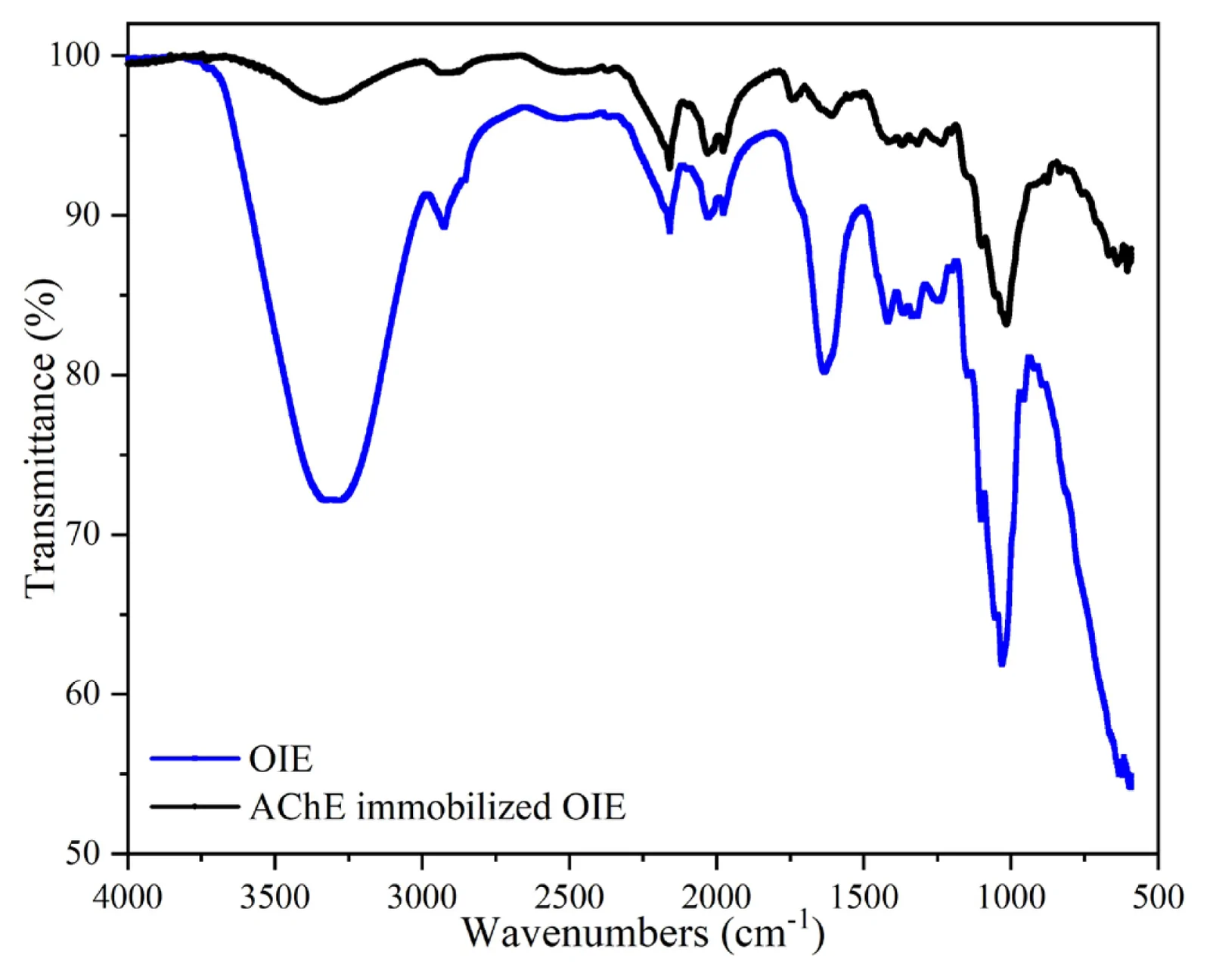
FTIR spectra of native and AChE-functionalized onion membranes.

The FTIR spectra of both native and AChE-immobilized onion membranes are displayed in Figure 1, providing insights into the structural and chemical modifications induced by the immobilization process. In the spectrum of the untreated membrane, a broad band observed in the range of 3200–3600 cm^−1^ is attributed to O–H stretching vibrations, corresponding to hydroxyl functionalities present in cellulose microfibrils [38].

Following enzyme immobilization, this band exhibits a slight shift along with a reduction in intensity, suggesting the involvement of hydroxyl groups in noncovalent interactions, such as hydrogen bonding, with acetylcholinesterase molecules. The enzyme bound membrane displays characteristic peaks at around 1650 cm^−1^ and 1550 cm^−1^, corresponding to amide I (C=O stretching) and amide II (N-H bending) bands, respectively [39]. These protein-related absorptions indicate the presence of AChE on the membrane surface, as they are not observed in the control spectrum. In the spectral region between 1000 and 1300 cm^−1^, associated with C–O stretching and C–O–C glycosidic linkages within the polysaccharide framework [39], subtle changes in peak intensities and slight shifts are observed after immobilization, suggesting possible interactions or conformational adjustments in the cellulose matrix. Additionally, a general reduction in overall transmittance is observed in the spectrum of the immobilized membrane, which may be attributed to increased molecular density upon enzyme association. Overall, the FTIR results support the presence of AChE on the onion membrane and suggest interactions between the enzyme and functional groups of the support material. However, it should be noted that FTIR analysis alone is not sufficient to fully elucidate the exact nature of the enzyme– support interactions, and the results should be interpreted in conjunction with complementary techniques such as SEM.

### 3.3. SEM analysis

**Figure 2:**
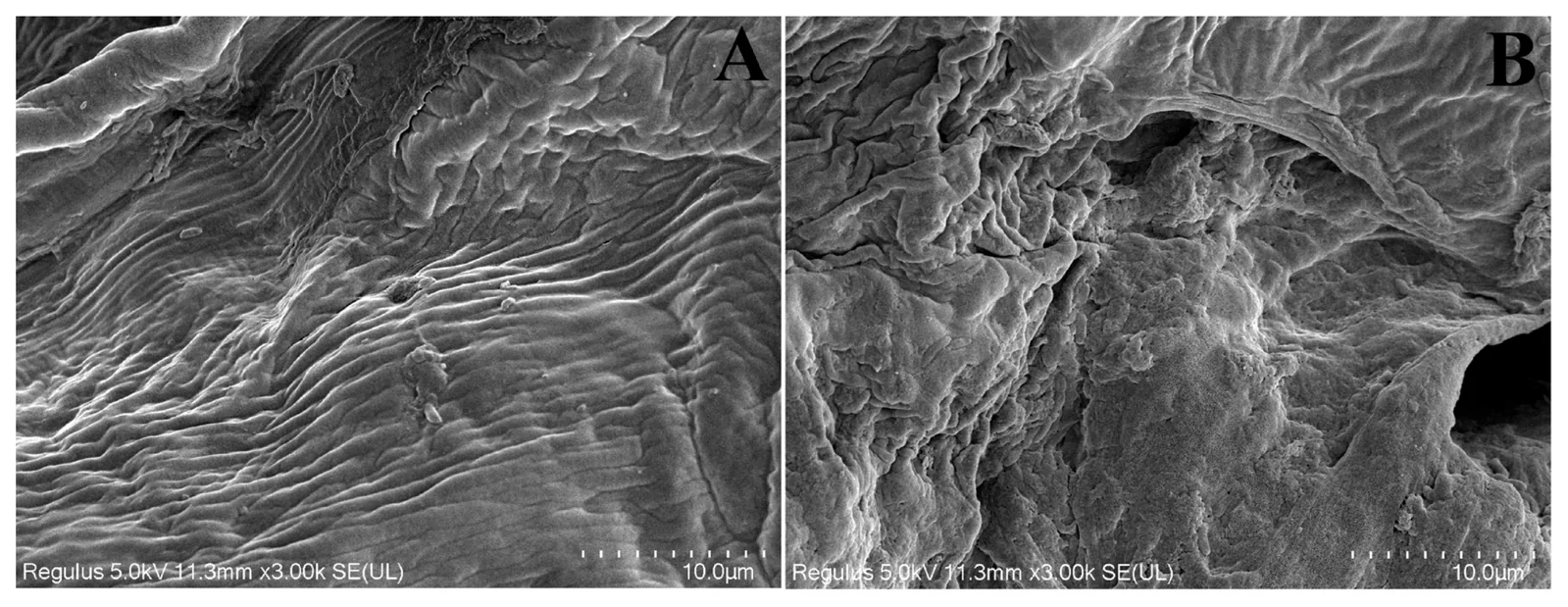
SEM images of (A) native and (B) AChE-immobilized onion membrane.

SEM was employed to evaluate morphological alterations in the onion membrane surface before and after AChE immobilization. Figure 2A illustrates that the native onion membrane exhibits a smooth and organized surface structure, characterized by uniform texture and parallel alignment of microfibrils. The observed surface consists of organized ridges and grooves, which are characteristic of the natural cellulose-based structure of onion membrane. The observed smoothness and orderly microfibrillar arrangement in the native membrane imply a favorable surface topology for effective enzyme adsorption. In comparison, the SEM micrograph of the enzyme-immobilized onion membrane (Figure 2B) reveals distinct morphological alterations relative to the untreated sample. The smooth and organized structure of onion membrane has become rougher, with the appearance of irregular, aggregated, and denser surface regions. These changes are indicative of successful AChE immobilization, as the enzyme molecules and crosslinking agents (e.g., glutaraldehyde) likely interact with the onion membrane surface, creating a more irregular texture. The observed roughness and deposition of enzyme molecules confirm that AChE has been effectively attached to the onion membrane surface through adsorption and cross-linking processes. The morphological alterations, such as increased surface irregularity and the presence of aggregated enzyme clusters, provide strong evidence of AChE attachment to the onion membrane carrier. This roughened surface enhances enzyme stabilization and reduces desorption, potentially contributing to improved catalytic performance and reusability. Comparison of Figures 2A and 2B demonstrates the successful immobilization of AChE onto onion membrane, as evidenced by the transformation of the smooth, organized control surface (onion membrane) into a rougher, denser structure postimmobilization. These structural modifications confirm the effectiveness of the immobilization process and emphasize the potential of onion membrane as a natural carrier for enzyme immobilization.

### 3.4. Temperature properties

**Figure 3:**
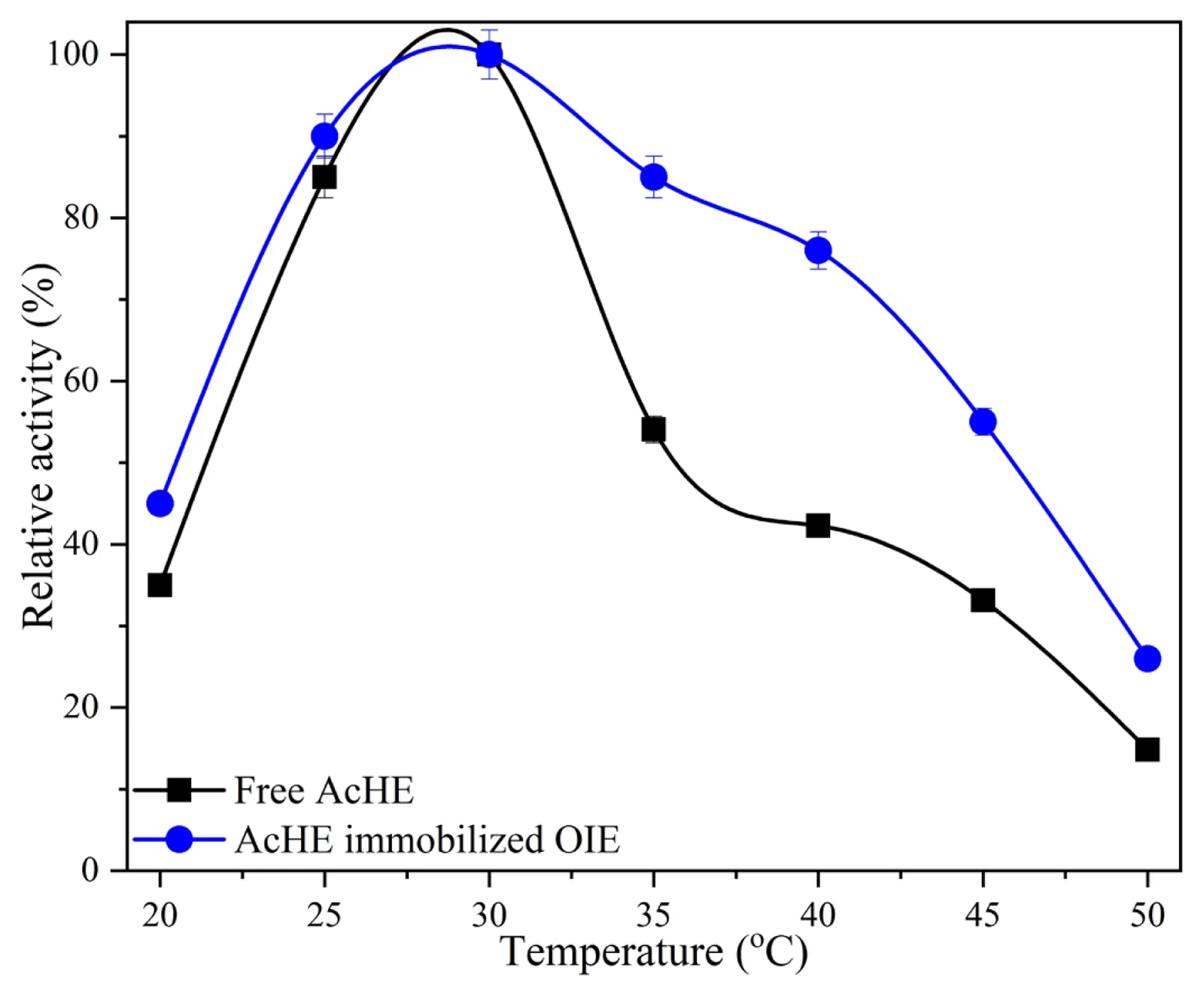
Temperature-dependent activity profiles of free AChE and AChE immobilized on onion membrane.

The optimal temperature identified for free AChE and AChE immobilized on onion membrane was 30 °C, as shown in Figure 3. At this temperature, both forms of the enzyme displayed their maximum relative activity. While immobilization did not alter the optimum temperature of AChE, it significantly improved the enzyme’s thermal stability at temperatures above 30 °C. The immobilized AChE demonstrated superior thermal stability compared to its free counterpart across the temperature range tested. At 40 °C, the immobilized form retained nearly 80% of its initial activity, whereas the free enzyme maintained only about 50%. This trend became more pronounced at elevated temperatures; at 45 °C, the activity of the immobilized enzyme dropped to approximately 60%, while that of the free enzyme fell below 30%. By 50 °C, the free form exhibited an almost complete loss of activity, in contrast to the immobilized enzyme, which still preserved roughly 30% of its catalytic function. These results indicate that while immobilization on onion membrane did not alter the enzyme’s optimal temperature, it conferred enhanced thermal stability by protecting the enzyme from inactivation at elevated temperatures. The onion membrane likely stabilized the enzyme’s conformation, reducing thermal denaturation and preserving enzymatic functionality under heat stress. In a study involving the immobilization of AChE onto polyacrylic acid/multiwalled carbon nanotube nanofibers, the free enzyme exhibited an optimum temperature of 40 °C, whereas the immobilized form demonstrated a shift in optimum temperature to 45 °C [40]. In another study involving AChE immobilization on polyacrylic acid-based nanofibers, the optimum temperatures reported for free and immobilized AChE were 30 °C and 35 °C, respectively. However, it was noted that, at all temperatures tested (20–40 °C) except 35 °C, the free AChE exhibited higher activity compared to the immobilized form [41].

**Figure 4:**
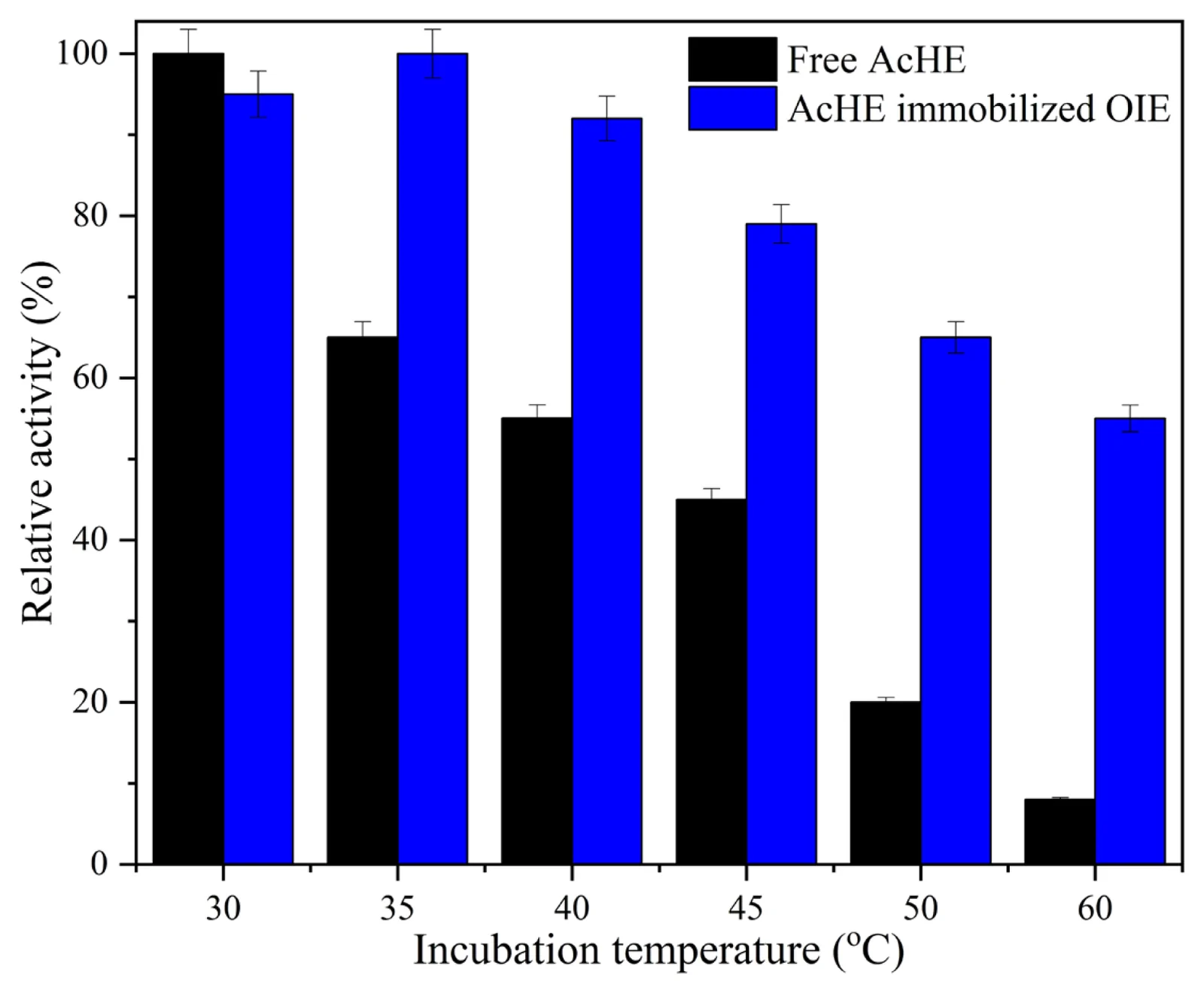
Comparison of thermal stability between free and immobilized AChE.

The thermal stability of free AChE and AChE immobilized on onion membrane was evaluated by incubating the enzymes at temperatures ranging from 30 °C to 60 °C for 1 h, followed by measurement of their relative activities (Figure 4). The results clearly indicate that immobilization significantly improves the thermal resistance of AChE. At lower temperatures (30–35 °C), both free and immobilized AChE retained over 90% of their initial activity. However, with increasing temperature, the activity of free AChE decreased sharply, retaining only 45% at 40 °C and dropping below 10% at 60 °C. In contrast, AChE immobilized on onion membrane preserved substantially higher activity under the same conditions, retaining approximately 85% at 40 °C, 78% at 45 °C, and 60% at 50 °C. Even at 60 °C, the immobilized enzyme maintained nearly 40% of its initial activity, while the free enzyme was almost completely inactivated. The improved thermal stability of immobilized AChE can be explained by considering the microstructural characteristics of the onion membrane, as evidenced by SEM analysis. The transition from a smooth and organized surface to a rougher and denser morphology after immobilization suggests the formation of a heterogeneous microenvironment that can physically confine enzyme molecules. This confinement effect limits molecular mobility and reduces the extent of thermally induced conformational changes. Furthermore, the porous and cellulose-based nature of the onion membrane likely supports the retention of a hydration layer around the enzyme, which is essential for maintaining its native conformation under thermal stress. In addition, glutaraldehyde-mediated cross-linking introduces covalent stabilization, increasing structural rigidity and preventing unfolding at elevated temperatures. The combined effects of physical confinement, intermolecular interactions, and covalent cross-linking contribute to the enhanced resistance of immobilized AChE to thermal denaturation.

Similar improvements in thermal stability upon immobilization have been reported in the literature. For instance, in a study in which AChE was immobilized onto Fe_3_O_4_@MXene@PEI aerogel, the free enzyme exhibited increasing catalytic activity between 25 and 50 °C, while the immobilized form reached maximum activity at 40 °C and remained stable at higher temperatures [33]. Additionally, AChE immobilized on styrene maleic anhydride nanofibers retained 55% of its activity after 60 min at 50 °C, corresponding to a 45% loss in activity [42].

### 3.5. pH properties

**Figure 5:**
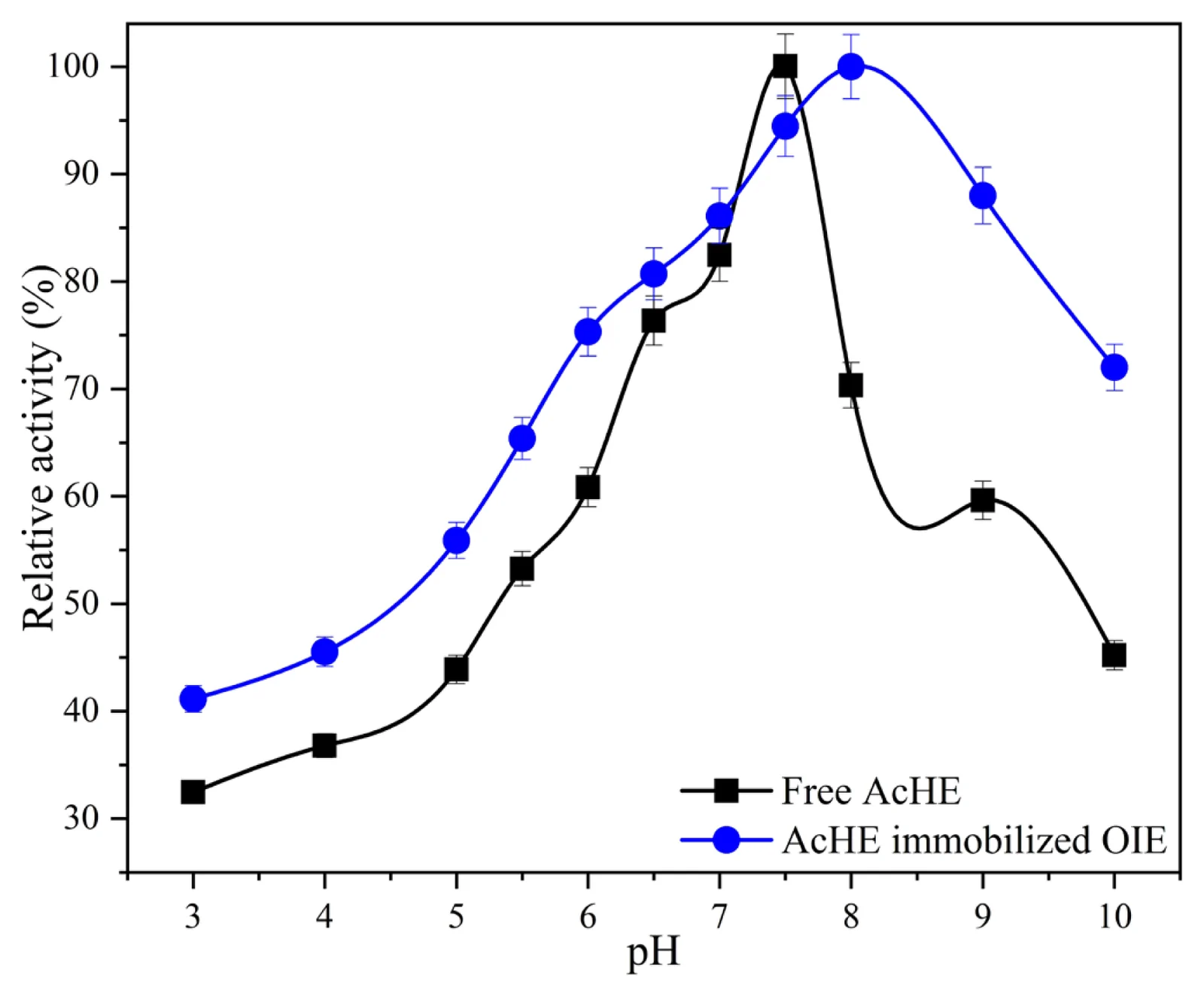
Optimum pH profiles of free AChE and AChE immobilized on onion membrane.

As seen in Figure 5, AChE exhibited maximum catalytic activity at pH 7.5, indicating a preference for neutral conditions. In contrast, AChE immobilized on onion membrane demonstrated peak activity at pH 8.0, suggesting that the immobilization process altered the enzyme’s microenvironment in a way that favored slightly alkaline conditions. Outside their respective optimum pH values, both enzyme forms showed reduced relative activity. However, AChE immobilized on onion membrane retained significantly higher activity over a broader pH range. At pH 5.0, the immobilized enzyme retained approximately 60% of its initial activity, whereas the free AChE retained only 45%. Similarly, at pH 9.0, the immobilized AChE maintained about 80% activity, while the activity of the free enzyme declined to 50%. This enhanced pH tolerance of the immobilized enzyme is likely due to the protective and buffering effects of the onion membrane support, which helps stabilize the enzyme’s tertiary structure and mitigates pH-induced denaturation.

Consistent with these findings, in various studies similar shifts in the optimum pH following enzyme immobilization have been reported. For instance, Çakıroğlu et al. reported a shift in the optimum pH from 7.5 (free AChE) to 8.0 upon immobilization on polyacrylic acid-based nanofibers [41]. Likewise, in our previous work, the optimum pH found for free AChE was 7.0, while AChE immobilized on Zn^2+^-doped PVA nanofibers showed an expanded optimum pH range of 7.5–8.5 [30].

**Figure 6:**
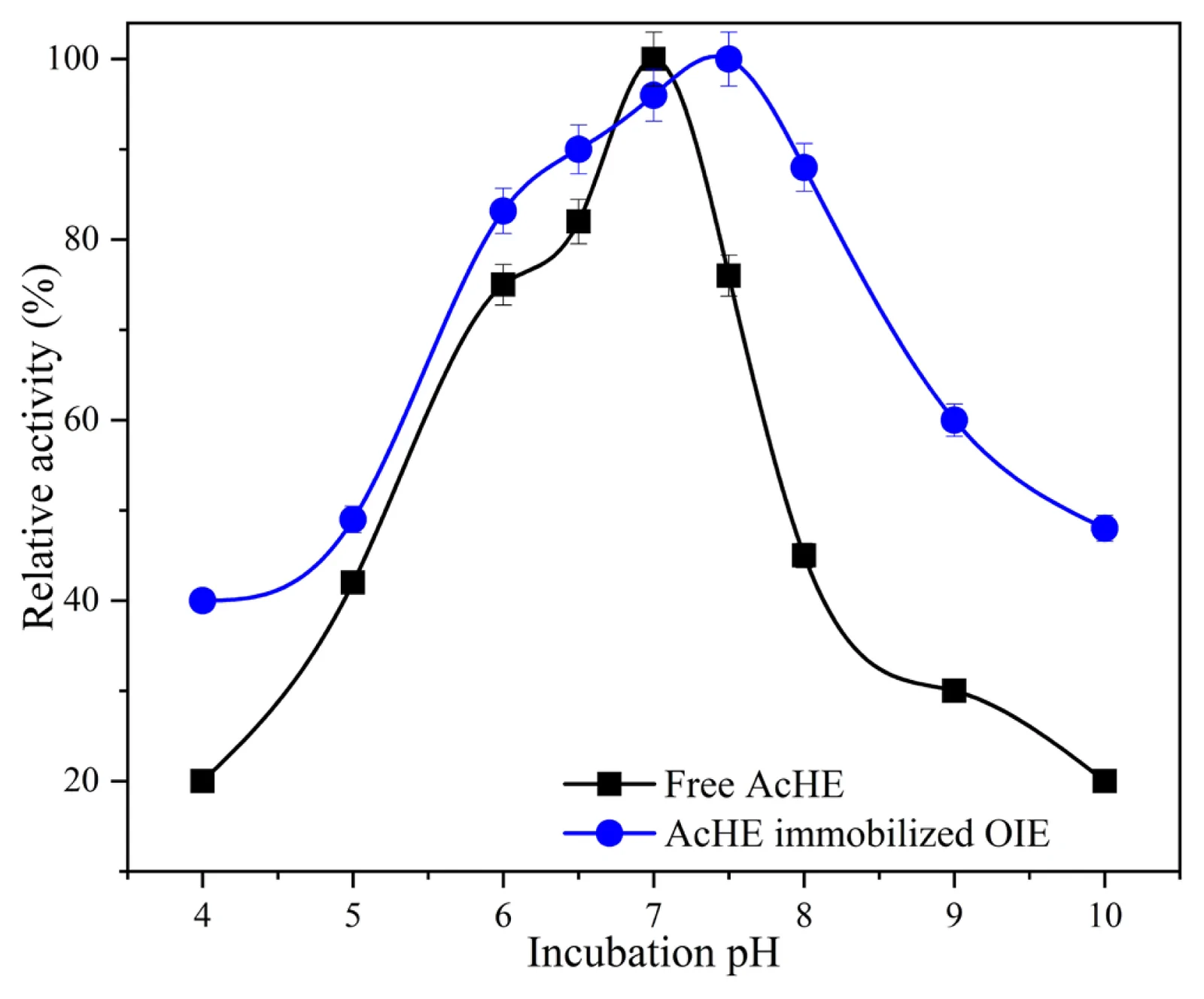
pH stability profiles of free AChE and AChE immobilized on onion membrane.

The pH stability of free AChE and AChE immobilized on onion membrane was evaluated by incubating the enzymes in buffer solutions ranging from pH 4.0 to 10.0 for 1 h, followed by activity measurements (Figure 6). The results demonstrate that immobilization significantly enhanced the enzyme’s stability across a broader pH range compared to its free form. Free AChE displayed maximum stability near its optimal pH of 7.0, but its activity declined rapidly under both acidic and alkaline conditions. At pH 5.0, the free enzyme retained only about 40% of its initial activity, and this value dropped below 20% at pH 9.0. In contrast, AChE immobilized on onion membrane retained considerably higher activity throughout the pH range tested, maintaining approximately 60% of its activity at pH 5.0 and around 80% at pH 9.0. This indicates that immobilization conferred enhanced tolerance to both acidic and basic environments. The improved pH stability of the immobilized enzyme can be attributed to the microstructural characteristics of the onion membrane, as supported by SEM observations. The roughened and heterogeneous surface formed after immobilization suggests the development of a localized microenvironment around the enzyme molecules. This microenvironment can partially buffer external pH fluctuations by modulating the immediate ionic conditions surrounding the enzyme. Additionally, the cellulose-rich and hydrophilic nature of the onion membrane may facilitate the retention of a hydration shell, which helps preserve the native conformation of the enzyme under extreme pH conditions. The porous architecture of the membrane likely contributes to partial entrapment of enzyme molecules, thereby limiting their exposure to drastic pH changes and reducing conformational disruption. Furthermore, glutaraldehyde-mediated cross-linking enhances structural rigidity, making the enzyme less susceptible to pH-induced denaturation. These combined effects result in a more stable catalytic system across a wider pH range.

Comparable observations were reported in a study involving AChE immobilized on Fe_3_O_4_@MXene@PEI aerogel, in which the immobilized enzyme exhibited enhanced stability in the pH range of 5.0–7.0. However, at higher pH values (8.0–10.0), a gradual decline in activity was noted. Specifically, at pH 9.0, the free enzyme lost approximately 70% of its activity, while the immobilized form retained somewhat higher stability but still experienced a nearly 60% loss [33].

### 3.6. Reusability of AChE immobilized on onion membrane

**Figure 7:**
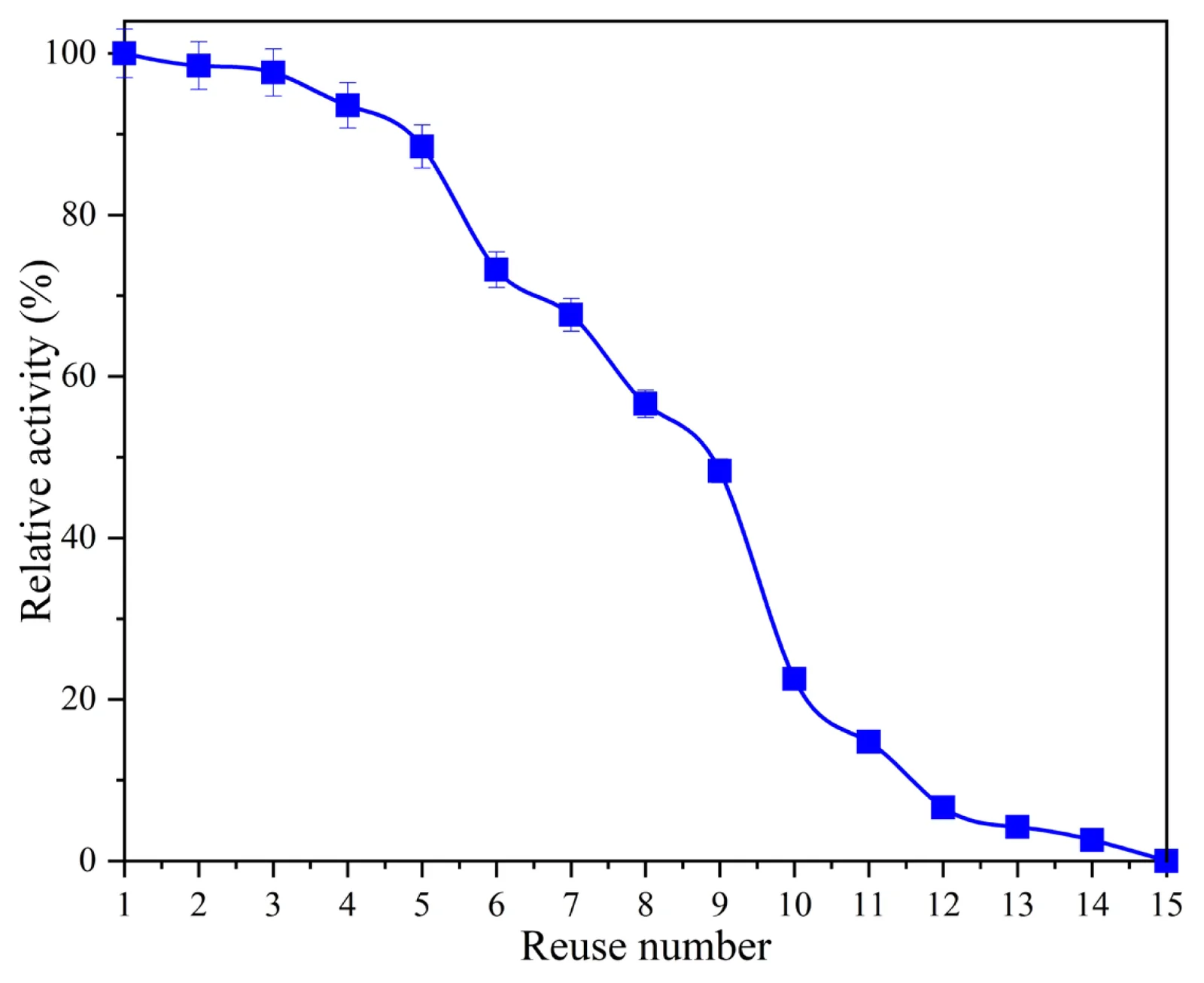
The reusability of AChE immobilized on onion membrane.

The reusability of AChE immobilized on onion membrane was evaluated over 15 consecutive catalytic cycles by measuring its relative enzymatic activity after each use (Figure 7). The results revealed a gradual decline in activity with successive cycles; however, a substantial portion of the enzyme’s activity was retained throughout the reuse period. This performance underscores the operational stability and durability of the immobilized enzyme system. After five cycles, the enzyme retained approximately 95% of its initial activity, indicating minimal loss in catalytic performance. As the number of cycles increased, the activity began to decline more noticeably. By eight cycles, AChE immobilized on onion membrane maintained around 60% of its activity, reflecting the cumulative impact of repeated reaction cycles on enzyme stability. Beyond eight cycles, the rate of activity loss became more pronounced. After 10 cycles, the activity dropped to approximately 45% and, by 15 cycles, the enzyme retained only 5% of its initial activity. The gradual loss of activity is likely attributable to a combination of factors, including partial desorption of the enzyme from the onion membrane surface, conformational alterations induced by repeated substrate exposure, and possible inactivation during successive reaction and washing steps. Similar declines in activity have been widely reported in enzyme immobilization systems and are generally associated with cumulative operational stress [17,30]. Importantly, the progressive nature of activity decline, rather than an abrupt decrease, suggests that the onion membrane largely maintains its structural integrity throughout the reuse cycles. No visible disintegration or loss of membrane structure was observed during the experimental process, indicating that the support material remains physically stable under repeated reaction and washing conditions. This sustained catalytic activity over multiple cycles indirectly supports the durability of the onion membrane. However, the significant reduction in activity after extended reuse cycles highlights a limitation of the system, which is primarily associated with enzyme-related factors rather than substantial degradation of the support material.

Following 10 repeated cycles of use, AChE immobilized on styrene–maleic anhydride electrospun mats maintained nearly 60% of its original catalytic activity, indicating moderate reusability [42]. In a previous study we conducted, AChE immobilized on Zn^2+^-doped PVA nanofibers was found to lose more than 50% of its activity after 12 consecutive uses [30]. AChE immobilized on MnO_2_-ZIF-67 retained 78.2% of its initial activity after five consecutive catalytic cycles, indicating good reusability and operational stability [43].

### 3.7. Storage stability of free AChE and AChE immobilized on onion membrane

**Figure 8:**
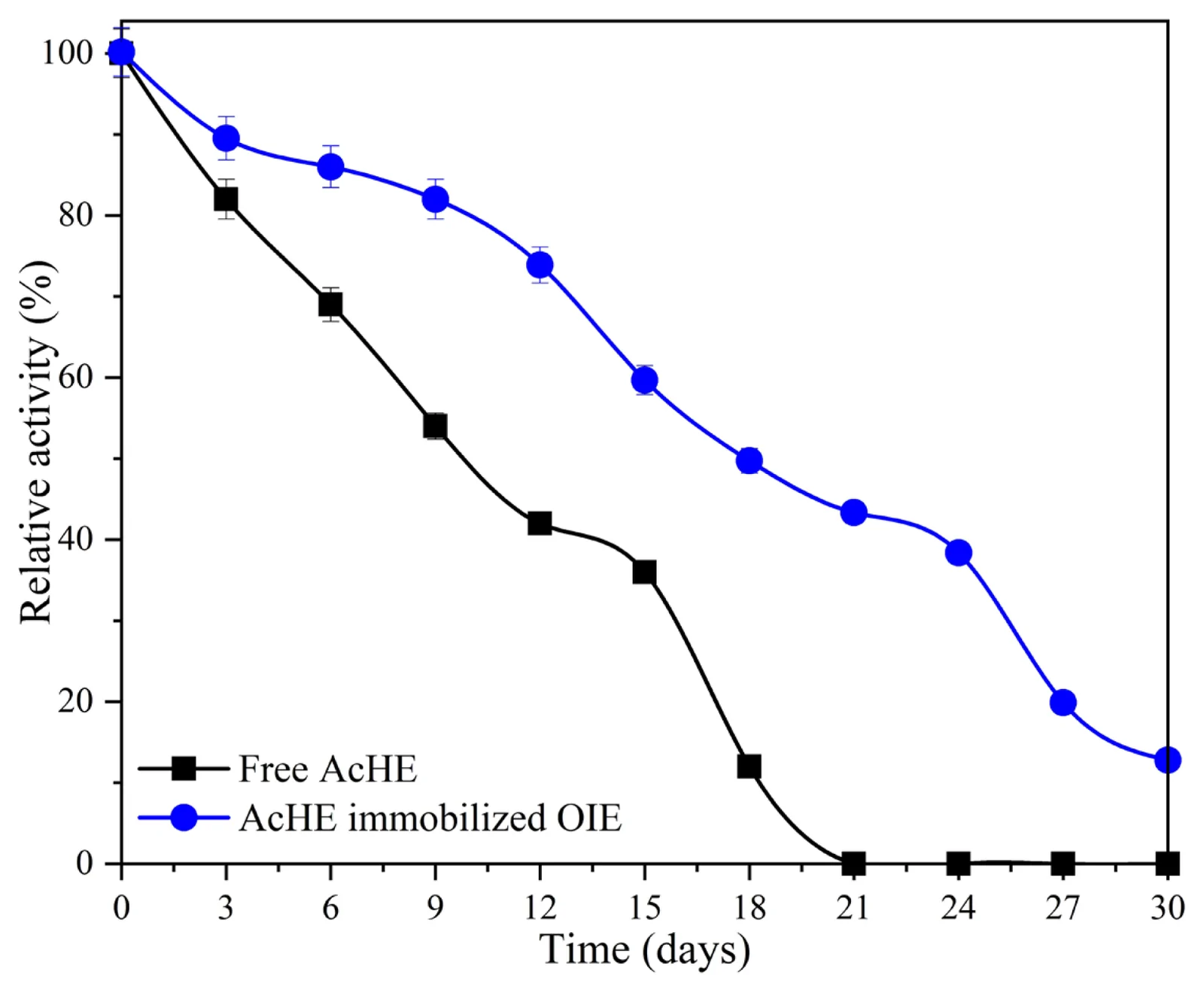
Storage stability properties of free AChE and AChE immobilized on onion membrane.

The storage stability of free AChE and AChE immobilized on onion membrane was evaluated over a 30-day period at 25 °C, and the results are presented in Figure 8. The data clearly demonstrate a substantial improvement in the long-term stability of the immobilized enzyme compared to its free counterpart. Free AChE exhibited a rapid loss of activity within the first 2 weeks of storage, retaining only 40% of its initial activity by day 15. By day 18, the free enzyme had completely lost its catalytic function, likely due to structural denaturation or aggregation caused by prolonged exposure to ambient conditions. In contrast, AChE immobilized on onion membrane displayed a more gradual decline in enzymatic activity. After 15 days, the immobilized enzyme retained approximately 60% of its initial activity, significantly outperforming the free enzyme under identical conditions. Even at the end of the 30-day storage period, the immobilized AChE preserved around 20% of its original activity, whereas no detectable activity remained for the free form. The enhanced storage stability of the immobilized enzyme can be explained by the combined effects of the onion membrane microstructure and the immobilization strategy. As suggested by SEM analysis, the roughened and heterogeneous surface formed after immobilization likely creates a confined microenvironment that protects enzyme molecules from external stress factors during storage. This microenvironment may help maintain a stable hydration layer around the enzyme, which is essential for preserving its native conformation over time. Additionally, the cellulose-rich and hydrophilic nature of the onion membrane can facilitate favorable enzyme support interactions, reducing conformational flexibility and limiting aggregation. The porous architecture of the membrane may also contribute to partial physical entrapment, minimizing enzyme desorption during prolonged storage. Furthermore, glutaraldehyde-mediated cross-linking enhances structural rigidity, making the enzyme less susceptible to long-term denaturation. These combined factors result in a slower rate of activity loss for the immobilized enzyme and indicate that the onion membrane provides a stable and protective support environment during extended storage. While minor structural or surface-level changes in the membrane over time cannot be completely excluded, the results clearly demonstrate that the primary contribution to enhanced storage stability arises from the protective microenvironment and immobilization-induced stabilization effects.

When compared with previously reported immobilization platforms, the onion membrane-based system demonstrates several notable advantages as well as certain limitations. Synthetic supports such as nanoparticles, nanofibers, and aerogels often provide high surface area and tunable properties; however, they typically involve complex fabrication procedures, higher costs, and potential environmental concerns. In contrast, the onion membrane used in the present study represents a naturally abundant, biodegradable, and low-cost alternative that does not require extensive chemical modification. In terms of performance, the immobilized AChE in our study exhibited enhanced thermal and pH stability and maintained considerable activity over multiple reuse cycles, which is comparable to or, in some cases, superior to systems based on synthetic supports. However, similar to many immobilized enzyme systems, a reduction in catalytic activity and substrate affinity was observed, highlighting a common trade-off between stability and catalytic efficiency. Overall, while advanced materials may offer higher surface area or more controlled functionalization, the onion membrane provides a balanced combination of simplicity, sustainability, and functional performance. These characteristics make it a promising alternative support material, particularly for applications in which cost-effectiveness and environmental compatibility are of primary importance.

### 3.8. Comparative kinetic analysis of free and immobilized AChE

The kinetic behavior of free AChE and AChE immobilized on onion membrane was investigated by determining their Michaelis–Menten constant (*K*_m_) and maximum reaction velocity (*V*_max_) to evaluate the effect of immobilization on enzymatic performance. For free AChE, *K*_m_ was 0.142 mM and *V*_max_ was 1.09 mmol/min. Upon immobilization onto onion membrane, the enzyme exhibited a slightly increased *K*_m_ of 0.159 mM and a higher *V*_max_ of 1.48 mmol/min. The increase in *K*_m_ indicates a reduction in substrate affinity, which is a commonly observed limitation in immobilized enzyme systems. This effect can be attributed to mass transfer resistance, as the substrate must diffuse through the support matrix to reach the active site, as well as steric hindrance and possible conformational constraints imposed by the immobilization process. Similar increases in *K*_m_ values have been widely reported for AChE immobilized on various supports [30,41,44]. Moreover, the observed increase in *V*_max_ suggests that immobilization may enhance the catalytic turnover rate under the conditions tested. This improvement can be associated with stabilization of the enzyme structure, reduced aggregation, and a more favorable microenvironment provided by the support matrix. Overall, the kinetic results highlight a typical trade-off in immobilized enzyme systems: while substrate affinity is slightly reduced, catalytic efficiency and operational stability are improved. Although the increase in *K*_m_ represents a limitation of the system, the enhanced *V*_max_ and improved stability indicate that the immobilized enzyme remains suitable for practical applications.

## 4. Conclusions

In the present study, AChE was successfully immobilized onto onion membrane through a combination of adsorption and cross-linking techniques. Optimization of the immobilization parameters demonstrated that onion membrane serves as an effective and sustainable support material for enzyme immobilization. Characterization analyses, including SEM and FTIR, confirmed the structural interactions between AChE and the onion membrane surface, validating the efficiency of the immobilization process. The immobilized AChE demonstrated improved thermal and pH stability relative to the free enzyme, retaining higher catalytic activity across a broader range of operational conditions. Both free and immobilized AChE exhibited an optimum temperature of 30 °C. However, the immobilized enzyme retained significantly higher catalytic activity at elevated temperatures, maintaining approximately 80% of its activity at 40 °C. In addition to improved thermal stability, the immobilized AChE also demonstrated superior operational reusability, retaining over 60% of its initial activity after 10 consecutive cycles. Furthermore, the enzyme showed enhanced storage stability, preserving around 40% of its original activity after 30 days of storage at 25 °C. The enhanced stability and performance of the immobilized enzyme are attributed to the presence of functional groups such as hydroxyl, carboxyl, and phenolic moieties on the surface of the onion membrane. These groups facilitate stable enzyme attachment and promote strong interactions between AChE and the carrier material, thereby improving compatibility and preserving enzyme functionality under repeated or prolonged usage. When compared with conventional immobilization platforms such as nanoparticles, nanofibers, and aerogels, the onion membrane offers a distinct advantage in terms of cost-effectiveness, biodegradability, and ease of preparation, while still providing competitive stability and reusability. Although a moderate decrease in catalytic activity and substrate affinity was observed, this trade-off is consistent with commonly reported immobilization systems. Overall, the improved stability, reusability, and simplicity of the immobilization process highlight the potential of onion membrane-immobilized AChE as a sustainable and efficient alternative for industrial and diagnostic applications, particularly in biosensors, neurodegenerative disease research, and pesticide detection systems.

## Figures and Tables

**Table 1:** Effects of key parameters on the immobilization efficiency of AChE using onion membrane.

Optimization parameters of AChE immobilization	Values	Specific activity of immobilized AChE (× 10^−3^ U/mg protein)

Amount of AChE (× 10^−4^ mg/mL)	2.5	0.71
	5.0	0.92
	7.5	1.06
	10.0	1.45
	**12.5**	**1.70**
	15.0	1.52

The quantity of onion membrane (mg)	5	0.78
	7.5	1.12
	10	1.57
	**12.5**	**2.88**
	15	1.51

Adsorption time (min)	10	0.69
	**15**	**1.27**
	20	0.79
	25	0.59
	30	0.34

GA concentration (%)	1	0.74
	2	0.88
	**3**	**1.10**
	4	0.44
	5	0.20

**Table 2:** Comparative activity parameters of free AChE and AChE immobilized on onion membrane.

	Activity (× 10^−6^ U)	Protein amount (× 10^−4^ mg)	Specific activity (×10^−3^ U/mg protein)	Bounded protein (%)	Specific activity yield (%)
Free AChE	3.50	10.50	3.33	-	100
AChE immobilized onion membrane	1.26	4.42	2.85	42.09	85.59
